# Probing behavior of the corn leafhopper *Dalbulus maidis* on susceptible and resistant maize hybrids

**DOI:** 10.1371/journal.pone.0259481

**Published:** 2022-05-31

**Authors:** Pablo Carpane, María Inés Catalano

**Affiliations:** 1 Bayer CropScience, Fontezuela, Buenos Aires, Argentina; 2 Centro de BioInvestigaciones (Universidad Nacional del Noroeste de la Provincia de Buenos Aires-CICBA), Pergamino, Buenos Aires, Argentina; 3 Centro de Investigaciones y Transferencias del Noroeste de la Provincia de Buenos Aires (CITNOBA-CONICET), Pergamino, Buenos Aires, Argentina; University of Saskatchewan College of Agriculture and Bioresources, CANADA

## Abstract

The corn leafhopper *Dalbulus maidis* is the main vector of the pathogens that cause corn stunt, a major disease of maize in the Americas. In line with plant resistance being an efficient tool to control diseases, the findings of a previous work showed that some corn hybrids are resistant to *D*. *maidis*. In this work, we assessed the probing behavior of *D*. *maidis* on susceptible and resistant corn hybrids using EPG (Electrical Penetration Graph) technology. Feeding of fifteen-day-old, non-inoculative females was recorded for 20 hours, with access to hybrids DK390, DK670, DK79-10, and DK72-10. Compared to the susceptible hybrid DK670, the other hybrids shifted *D*. *maidis* probing behavior in a way consistent with plant resistance to insects. This shift consisted of a higher number of probes of short duration, difficulties in attaining phloem ingestion and increase in xylem ingestion. In addition to this common shift in probing behavior, a phloem-located resistance factor was inferred in DK72-10 based on the longer time spent in phloem conditioning to attain phloem ingestion. In contrast, DK390 expressed the highest level of mesophyll and phloem-based resistance, in both cases seen with repeated attempts of short duration, a behavior typically associated with failed attempts to ingest. These findings support and are consistent with previous research, providing useful information to characterize maize hybrids resistant to *D*. *maidis*, and consequently to corn stunt.

## Introduction

Corn stunt is one of the most significant diseases affecting maize crop due to its high potential to cause yield losses [1–3]. The increasing prevalence of corn stunt in the Americas [2–5] after its first detection [6, 7] is a major constraint for corn production. The mollicute *Spiroplasma kunkelii* Whitcomb is the most common pathogen associated with corn stunt, although Maize bushy stunt phytoplasma (MBSP) and Maize rayado fino virus (MRFV) might be found as well [3, 4, 8]. These pathogens are transmitted only by a few leafhopper species, especially *Dalbulus maidis* (DeLong), the main vector species [8–11] due to its distribution [8, 9, 11, 12] and transmission efficiency of *S*. *kunkelii* [13, 14].

One of the strategies to reduce yield losses resulting from corn stunt, as well as from other diseases, is the use of resistant corn genotypes [15–18]. In pathosystems involving insect vectors, plant resistance can be aimed either at the pathogen or at the insect vector [19, 20], with the latter expressed as reducing preference (antixenosis) or survival (antibiosis). These resistance traits are usually present in other pathosystems [20, 21], and corn hybrids eliciting this behavior in *D*. *maidis* have been already found [22]. Both resistance traits reduce the duration of insect-plant interaction [19–21], which in turn might reduce the inoculation efficiency of the pathogen *S*. *kunkelii*, which increases up to 80% if such interaction is extended from 1 to 48 h [14, 23].

However, the characterization of hybrids either triggering antibiosis or antixenosis fails to provide insights on the nature and location of plant resistance, hindering the development of breeding strategies like pyramiding resistance genes in search of durable resistance [18]. Because *S*. *kunkelii* resides in phloem [24], efficiency of inoculation and acquisition of *S*. *kunkelii* are directly related to the time spent by *D*. *maidis* respectively conditioning and ingesting in this tissue. Hence, a method capable of characterizing *D*. *maidis* feeding at plant tissue level would ease the identification and characterization of the effect of resistance genes, thus contributing to designing even more effective strategies to control corn stunt.

The study of insect of insect probing behavior using electrical penetration graph technology (also known as electropenetrography [25, 26], both abbreviated EPG) identifies the activities performed by insects while inserting their stylets into plant tissues. EPG can aid identification of the mechanism and location of the traits conferring plant resistance at the tissue level [25, 26]. This is particularly useful in pathosystems where the dynamics of pathogen transmission is highly related to the activities performed by insects in phloem [25, 27]. For instance, leafhoppers exposed to resistant genotypes typically spend less time probing into phloem than those probing on susceptible genotypes, decreasing the transmission efficiency of phloem-inhabiting pathogens [21, 28]. Also, factors conferring plant resistance can be located in other tissues [26], and so they may be encountered by insects before reaching phloem [26, 29]. Accordingly, the study of probing behavior contributes to understanding the basis of plant resistance in pathosystems involving vectors and may assist plant breeders in selecting and characterizing resistant genotypes [20, 30, 31].

In the case of *D*. *maidis*, previous work [27] has found that inoculation and acquisition of the pathogen *S*. *kunkelii* take place during phloem conditioning and ingestion respectively, which were in turn associated with specific waveforms in the study of the probing behavior of this insect species. Consequently, the objective of this work was to use this previous information to characterize the probing behavior of *D*. *maidis* adults with access to corn hybrids of different resistance to this insect species, to gain a better understanding of the mechanism and location of plant resistance traits in corn hybrids.

## Materials and methods

A colony of non-inoculative *D*. *maidis* was initiated from insects collected in Tucumán province, Argentina. This colony was reared on plants of the sweet corn variety “Maizón” at the CEBIO (BioResearch Center) of the UNNOBA-CICBA (National University of the North West of the Province of Buenos Aires—Scientific Research Commission of the Province of Buenos Aires), in Pergamino, Buenos Aires, Argentina. The colony was kept in aluminum-framed cages with a “*voile*” type nylon mesh, at a temperature of 25˚C, under a photoperiod of 16:8 (light: darkness) hours [13]. Four maize hybrids were used in this study: DK670 and DK72-10 from the temperate region of Argentina (where corn stunt is not present), and DK79-10 and DK390 from the tropical region of this country (where corn stunt is present). In a prior characterization of the resistance components of these hybrids to corn stunt [22], DK72-10 showed antibiosis and antixenosis to *D*. *maidis*, and DK390 antixenosis only. Moreover, DK79-10 and DK390 were found resistant to the pathogen *S*. *kunkelii*, and DK670 was considered susceptible to both *D*. *maidis* and *S*. *kunkelii*. The seeds used had no insecticides as part of the seed treatment. Plants were used at V3 (three leaves) stage.

Probing behavior of *D*. *maidis* was recorded using a Giga-8dd EPG-DC device (EPG Systems, Wageningen, The Netherlands) whose head stage amplifiers were placed inside of a Faraday cage (made of aluminum mesh and wooden frame) to reduce the interference of external electrical noise. The Giga 8dd had eight head state amplifiers, allowing testing of eight insect-hybrid combinations (two per hybrid) per recording session. Ten to fourteen-day-old, non-inoculative (as in [22]) *D*. *maidis* females were anesthetized by single confinement in glass tubes (1.5 x 15 cm), and ice cooled for 30 minutes. The immobilized insects were then gently placed on a Petri dish with the help of a small brush. The dorsal side of the scutellum was glued to a 12.5 μm diameter (sold as 0.0005 in), 2–3 cm long gold wire (Sigmund Cohn, Mount Vernon, NY) using silver conductive paint for electronic circuits (www.edelta.com.ar) under a binocular microscope. Neither vacuum nor CO_2_ were used for tethering. The other end of the gold wire was glued to a copper wire (around 0.5 mm diameter) soldered to a brass nail. The length and diameter of the gold wire allowed free movement of insects, and some of them even attempted to fly away before being placed in contact with the plants. After tethering, insects were starved for 1 hour before the EPG recording session began. Typically, 12–15 wires were mounted onto insects for each session; only those with insects moving steadily and so not damaged during tethering, were used for recordings. The electrode for the plant was a 2 mm diameter copper wire inserted into the moist soil of the pot that contained each plant. Plants were mounted by gently folding the newest expanded leaf around a 10 cm diameter cardboard cylinder and securing it with tape. The cylinder was then attached to a wooden stick inserted into the pot soil. This procedure exposed the abaxial side of the leaves to the insects and prevented the tissue exposed to the insect from moving during the EPG recording.

EPG recording sessions began by mounting one brass nail with wired insect into each of the eight head stage amplifiers of the EPG device and inserting the plant electrodes into the pot soil in such a way that insects and plants were two centimeters apart. Once all insects and plants were in place, the EPG controller was turned on and the pots were moved quickly (in less than 20 seconds) so the insects could reach the leaf tissue and start stylet probing. Initial controller settings were 50% gain with applied voltage and gain adjusted independently for each head stage amplifier for the first 30 minutes to ensure that the output voltage ranged from -5 to 5 V. The EPG-DC device has a fixed input resistance of 10^9^ Ω. In order to avoid differences in probing behavior that could be due to variations in the circadian cycle, recording sessions always started about 3:00 pm and lasted 20 h under continuous light, at a temperature of 25˚C and relative humidity close to 70%. Fifteen insect-plant replications per hybrid were recorded using new insects and plants in each replication. The head stage amplifier number assigned to each hybrid was randomized for each recording session.

**Fig 1:**
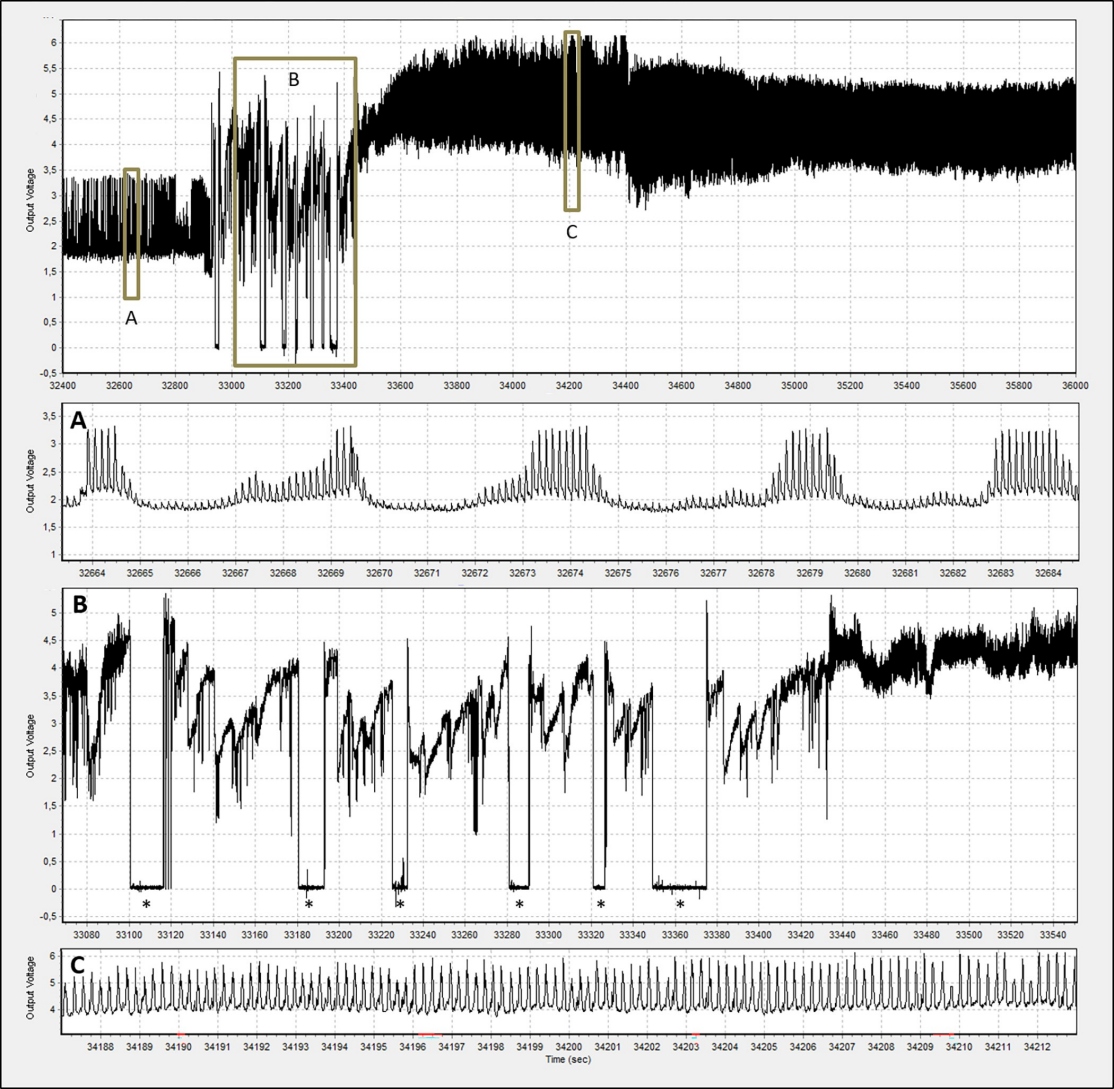
Samples of some major waveforms displayed during probing of *D*. *maidis* females. A: phloem ingestion (Dm5). B: a series of five short probes with stylet pathway (Dm1) waveforms only, combined with brief non-probing periods (NP) (*), followed by a longer probe in which the insect began to ingest from xylem (Dm2) (at around 33440 sec). C: xylem ingestion (Dm2). The X axis indicates the time (seconds) from the beginning of the recording session.

**Fig 2:**
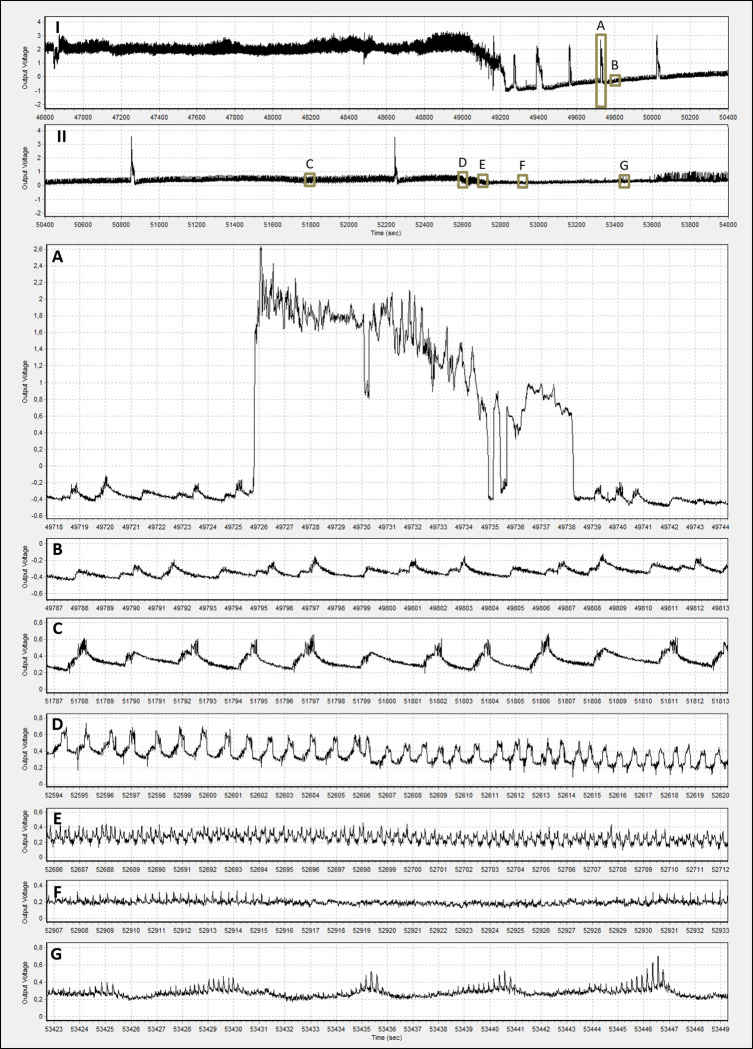
I and II: Progression of phloem conditioning (Dm4) to phloem ingestion (Dm5) of *D*. *maidis* insects with access to maize hybrids. Inlets: A: “spikes” of high amplitude and irregular shape. B-D: phloem conditioning. E: transition to phloem ingestion. F-G: phloem ingestion. The X axis indicates the time (seconds) from the beginning of the recording session.

EPG waveforms were acquired using Stylet+ software and stored in the hard drive of the computer. In this work, a “probe” was defined as a length of time in which the insects kept their stylets inserted into plant tissues, seen in the EPG device as a deviation from a flat line at 0 V when insect stylets were not inserted. In most probes, insects would often perform more than one activity, with each activity observable as a specific waveform recognized by the human eye. Each uninterrupted duration of a certain waveform was called a “waveform event”. The observed waveforms (Figs 1 and 2) matched closely those previously described for *D*. *maidis* [27], with insect activities described in Table 1. The *D*. *maidis* waveforms originally numbered 1, 2, 3, 4, 5, and 6 in [27] were here renamed Dm1, Dm2, Dm3, Dm4, Dm5, and Dm6 respectively, according to the nomenclature suggested recently [32, 33]. To ensure that the normally evolving phloem conditioning waveform found here matched the evolving Dm4 waveform described by Carpane et al. 2011 [27], raw waveforms from that study were recovered and shown in S1 Fig, confirming that they were similar and progressed over time in the same manner.

**Table 1:** Updated waveforms names / biological meanings according to Carpane et al. 2011 (ref no. 27 in this paper).

Waveform Name ^(#)^	Description	Insect Activity
Dm1	Stylet pathway	Intracellular penetration of stylets through epidermis, mesophyll/parenchyma cells
Dm2	Active ingestion	Ingestion from xylem vessel or mesophyll cells
Dm3	Stylet work	Insect moving while keeping stylets inserted
Dm4	Phloem conditioning	Combined or alternating salivation and likely egestion into phloem sieve elements, associated with inoculation of *S*. *kunkelii*
Dm5	Phloem ingestion	Passive ingestion of phloem sieve elements, associated with acquisition of *S*. *kunkelii*
Dm6	Oviposition	Oviposition

# Waveform name was obtained by adding the “Dm” prefix to the waveform name given in Carpane et al. 2011 [27], as it was suggested before for leafhoppers and planthoppers [32].

The sequence of waveforms was saved in ANA files in Stylet+, after assessing (identifying and measuring their time of occurrence) visually each waveform event. Dm1, Dm2, Dm3, Dm4, Dm5 and Dm6 waveforms were loaded in ANA files as C, G, Id11, E1, E2 and Id12, respectively. Several variables of probing behavior were calculated from ANA files using EPG- Calc 6.1.3 [34]. These variables were at level of probes: number (NPI, number of probes per insect) and duration (PDP: probing duration per probe), and also of waveform events, such as number (NWEI: number of waveform events per insect), total time (WDI: waveform duration per insect), and average duration (WDEI: waveform duration event per insect) of each waveform during the recording. Sequential variables like the time or number of events from the beginning of the experiment to a specific waveform were also calculated. To avoid adaptation of insects to probe on non-preferred hybrids while tethered [35], these variables were calculated over the first 10 hours of the recordings, ending artificially the last waveform events. Waveforms Dm3 (Stylet work) and Dm6 (oviposition) were not further analyzed due to their rare presence (less than 1% of the recording time).

Statistical analyses were performed using Infostat [36]. The number of waveform events was analyzed with a Mixed Model ANOVA using a Poisson distribution, and the remaining variables were analyzed using a gamma distribution. In all the cases, the hybrid was the fixed effect, and the date of the recording session was the random effect. The models were adjusted using the nnet package [37] of the R language [38]. The pairwise significance of the differences in means was analyzed using Fisher LSD test. Differences were considered significant at α = 0.05.

## Results

**Fig 3:**
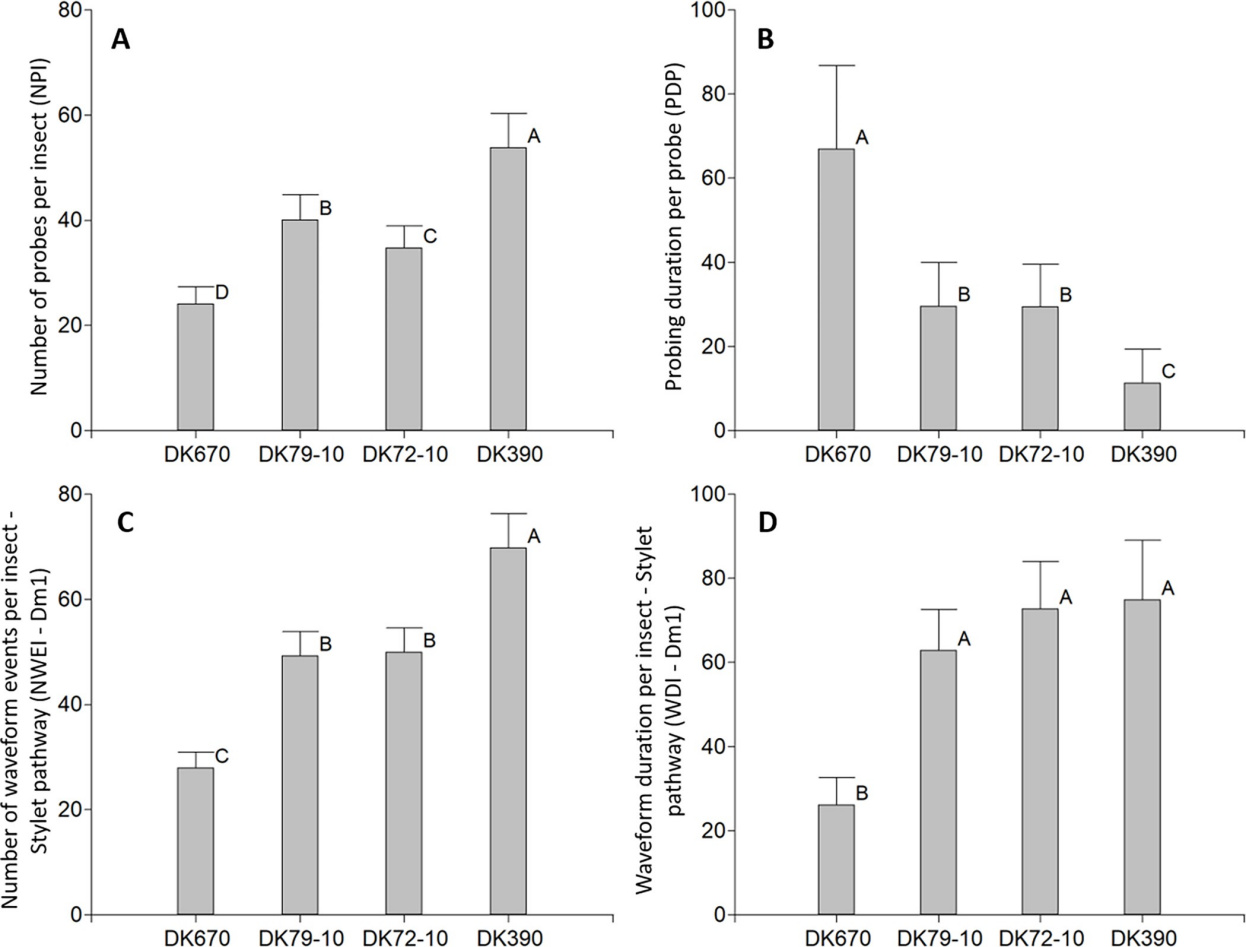
Variables of probing behavior of *D*. *maidis* with access to four maize hybrids, non-phloem phase. A: Number of probes per insect (NPI), B: Probing duration per probe (PDP), C: Number of waveform events per insect—Stylet pathway (NWEI—Dm1), D: Waveform duration per insect—Stylet pathway (WDI—Dm1). Values with the same letter are not significantly different according to contrasts in the Fisher LSD test (α = 0.05). Bars indicate standard error of the mean.

The probing behavior of *D*. *maidis* was highly dynamic towards the hybrids used, with the first probes occurring before the first minute, followed by a continuous succession of probes (mostly brief), with short non-probing periods, before insects began ingestion from xylem (described below). The Number of test probes, shorter than 3 minutes (NPI<3 min, Table 2) and the Number of probes per insect (NPI, Fig 3A, p<0.0001) were highest in DK390 and lowest in DK670. Conversely, the Probing duration per probe (PDP) was longest in DK670 and shortest in DK390 (Fig 3B, p<0.0001). In addition, the Number of waveform events—Stylet pathway (NWEI—Dm1, Fig 3C, p<0.0001) and Waveform duration per insect—Stylet pathway (WDI—Dm1, Fig 3D, p = 0.005) were lowest in DK670 and highest in DK390. In summary, while insects probing on DK670 had less, longer probes; those probing in DK390 showed more probes of short duration, most of them comprising only short periods of Dm1 (Stylet pathway) waveform.

**Table 2:** Variables (Mean +/- Standard Error [SE]) of probing behavior of *D*. *maidis* insects with access to four maize hybrids. Values with the same significance (Sig) letter are not significantly different according to contrasts in the Fisher LSD test (α = 0. 05).

Variable	p value	DK670			DK79-10			DK72-10			DK390		
		Mean	SE	Sig	Mean	SE	Sig	Mean	SE	Sig	Mean	SE	Sig
NPI<3 min	<0.0001	17.45	2.87	D	30.68	4.54	B	24.91	3.7	C	36.58	5.46	A
NPI>1stDm2	<0.0001	1.22	0.01	D	1.92	0.01	C	2.04	0.01	B	2.81	0.01	A
NWEI—Dm2	<0.0001	9.35	1.46	C	12.6	1.51	B	15.74	1.79	A	15.06	1.9	A
NWEI—Dm4	0.0038	1.8	0.51	C	3.59	0.72	B	3.69	0.64	B	5.79	0.99	A
t>1stDm4>1sDm5	0.0502	160.4	41.1	C	258.8	40.0	B	316.6	51.0	AB	390.8	74.3	A

Description of events of probing behavior: NPI<3 min: Number of test probes (shorter than 3 minutes), NPI>1stDm2: Number of probes per insect before first xylem ingestion, NWEI—Dm2: Number of waveform events per insect—Xylem ingestion, NWEI—Dm4: Number of waveform events per insect—Phloem conditioning, t>1stDm4>1stDm5: Time from first phloem conditioning to first phloem ingestion.

The time spent on xylem ingestion ranged from 50 to 100 min, around 8 to 16% of the length of the recording session (600 min). Xylem ingestion events occurred within the first stages of probing and within 5 hours of the last xylem ingestion event for insects unable to attain phloem ingestion. Half of the insects started xylem ingestion in less than 3 min, and by 10 min all insects had ingested from xylem. The only exception was one insect feeding on DK670, which started phloem conditioning at 3.5 min in the first probe and began phloem ingestion at 58 min in the third probe that lasted until the end of the recording session. In the comparison across hybrids, the Number of probes per insect before first xylem ingestion (NPI>1stDm2), and the Number of waveform events per insect—Xylem ingestion (NWEI—Dm2) were highest in DK390, lowest in DK670, and intermediate in the other hybrids (Table 2).

**Fig 4:**
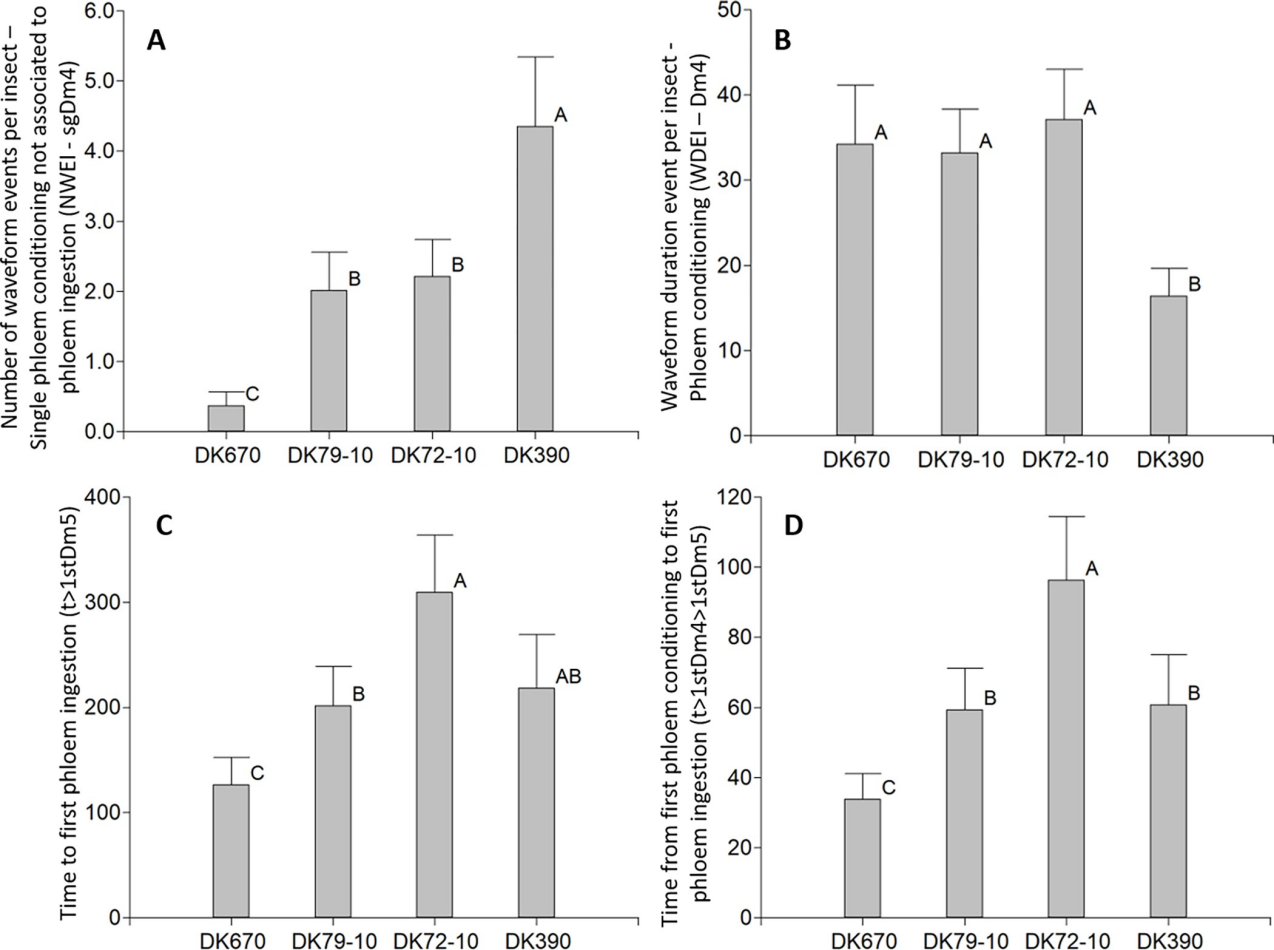
Variables of probing behavior of *D*. *maidis* insects with access to four maize hybrids, phloem phase. A: Number of waveform evens per insect—Single phloem conditioning not associated to phloem ingestion (NWEI—sgDm4), B: Waveform duration event per insect—Phloem conditioning (WDEI—Dm4), C: Time to first phloem ingestion (t>1stDm5), D: Time from first phloem conditioning to first phloem ingestion (t>1stDm4>1stDm5). Values with the same letter are not significantly different according to contrasts in the Fisher LSD test (α = 0.05). Bars indicate standard error of the mean.

Phloem conditioning took place later, after xylem ingestion. Half of the insects began phloem conditioning in less than two hours, with no differences across hybrids (results not shown). All the insects had at least one event of phloem conditioning. Both the Number of waveform events per insect—Phloem conditioning (NWEI—Dm4, Table 2), and the Number of waveform events per insect—Single phloem conditioning not associated to phloem ingestion (NWEI—sgDm4, Fig 4A, p = 0.0002) were higher in DK390, lowest in DK670 and intermediate in the other hybrids. On the contrary, the Waveform duration event per insect—Phloem conditioning (WDEI—Dm4) was shorter in DK390 than in the other hybrids (Fig 4B, p = 0.0038). In turn, both Time to first phloem ingestion (t>1stDm5, Fig 4C, p = 0.0034), and Time from first phloem conditioning to first phloem ingestion (t>1stDm4>1stDm5, Fig 4D, p<0.0001) were shortest in DK670, longest in DK72-10, and intermediate in the other hybrids.

**Fig 5:**
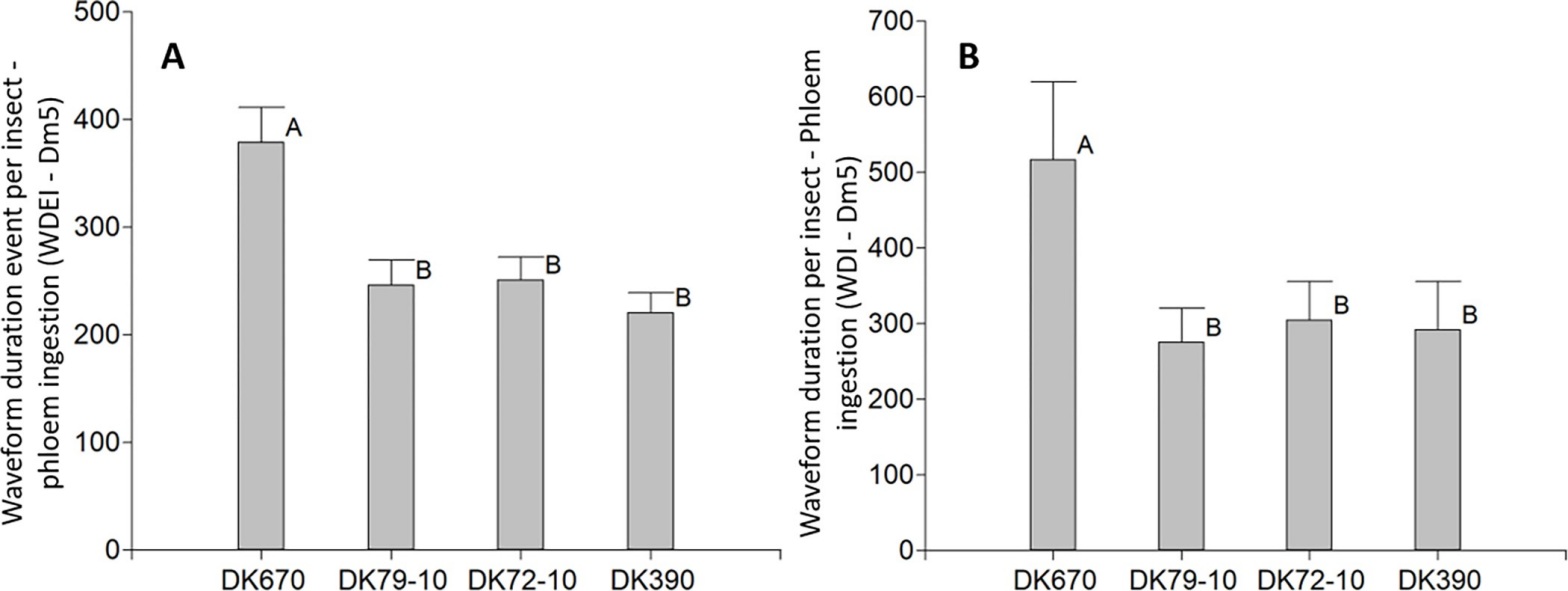
Variables of probing behavior by *D*. *maidis* insects with access to four maize hybrids, phloem ingestion. A: Waveform duration event per insect—Phloem ingestion (WDEI—Dm5), B: Waveform duration per insect—Phloem ingestion (WDI—Dm5). Values with the same letter are not significantly different according to contrasts in the Fisher LSD test (α = 0.05). Bars indicate standard error of the mean.

Phloem ingestion only occurred after the whole progression of the phloem conditioning waveform (Fig 2), was completed, which took place after two or three probes containing events of phloem conditioning interrupted at several stages. Phloem ingestion usually took place at late stages of insect-plant interaction, with half of the insects starting to ingest from phloem in about four hours, with no differences across hybrids (results not shown). All the insects in this study reached phloem ingestion at least once. While no differences were seen in the number of events of phloem ingestion (results not shown), the Waveform duration event per insect—Phloem ingestion (WDEI—Dm5, Fig 5A, p<0.0001) and the Waveform duration per insect—Phloem ingestion (WDI—Dm5, Fig 5B, p = 0.0107) were longer in DK670 than in the rest of the hybrids.

A summary of the variables or probing behavior related to plant resistance to *D*. *maidis* for the hybrids used is shown in Table 3. Compared to the susceptible hybrid DK670, insects probing on DK79-10 showed significant differences in several variables. Resistance located at Epidermis/Mesophyll was seen as an increase in the number of probes (both NPI<3 min and NPI), the number of events (NWEI) and total time (WDI) spent probing at these tissues (Dm1). Conversely, probes were shorter (PDP) in DK79-10 than in DK670. Insects probing on DK79-10 also required more probes to achieve xylem ingestion (NPI>1stDm2) and had more events of xylem ingestion (NWEI—Dm2). Phloem-located resistance in DK79-10 was seen as a higher number of events of phloem conditioning (NWEI—Dm4), specifically in those not associated to phloem ingestion (NWEI—sgDm4), a longer period from first phloem conditioning to first phloem ingestion (t>1stDm4>1stDm5), a delay in phloem ingestion (t>1stDm5), and a reduction in time spent in phloem ingestion (WDI—Dm5) due to a shorter phloem ingestion events (WDEI—Dm5). On top of the changes in probing behavior seen in DK79-10 compared to the susceptible DK670, both DK72-10 and DK390 differed from DK79-10 in several variables characterizing plant resistance. On one hand, insects probing on DK72-10 increased the number of xylem ingestion events (NWEI—Dm2) and delayed first phloem ingestion both from the first phloem ingestion (t>1stDm4>1stDm5) and from the start of the recording sessions (t>1stDm5), which indicate phloem-located resistance. On the other hand, insects probing on DK390 decreased probe duration (PDP) and increased the number of test (NPI<3 min) and total (NPI) probes and the number of events of stylet pathway (NWEI—Dm1), indicating epidermis/mesophyll-located resistance. A phloem-located resistance was also seen in DK390 as a higher number of events of phloem conditioning (NWEI—Dm4) of reduced length (WDEI—Dm4), being most of them not associated to phloem ingestion (NWEI—sgDm4). Additionally, the increase in the number of probes before first xylem ingestion (NPI<1stDm2) and in the number of events of xylem ingestion (NWEI—Dm2) in DK390 could be a consequence of resistance factors based either in epidermis/mesophyll or in phloem.

**Table 3:** Variables of probing behavior of *D*. *maidis* insects for hybrids showing significant differences from the susceptible hybrid DK670.

Location	Variable	Description	DK79-10	DK72-10	DK390
Epidermis/Mesophyll	NPI<3 min	Increase	X^#^	X	XX
Epidermis/Mesophyll	NPI	Increase	X	X	XX
Epidermis/Mesophyll	PDP	Decrease	X	X	XX
Epidermis/Mesophyll	NWEI—Dm1	Increase	X	X	XX
Epidermis/Mesophyll	WDI—Dm1	Increase	X	X	X
Xylem	NPI>1stDm2	Increase	X	X	XX
Xylem	NWEI—Dm2	Increase	X	XX	XX
Phloem	NWEI—Dm4	Increase	X	X	XX
Phloem	NWEI—sgDm4	Increase	X	X	XX
Phloem	WDEI—Dm4	Decrease			X
Phloem	t>1stDm4>1stDm5	Increase	X	XX	X
Phloem	t>1stDm5	Increase	X	XX	X
Phloem	WDEI—Dm5	Decrease	X	X	X
Phloem	WDI—Dm5	Decrease	X	X	X

# X: significantly different from DK670 according to Fisher LSD Tests. XX: significantly different from DK670 and DK79-10. NPI<3 min: number of brief probes (shorter than 3 minutes), NPI: Number of probes per insect, PDP: Probing duration per probe, NWEI—Dm1: Number of waveform events per insect—Stylet pathway, WDI—Dm1: Waveform duration per insect—Stylet pathway, NPI>1stDm2: Number of probes per insect before first xylem ingestion, NWEI—Dm2: Number of waveform events per insect—Xylem ingestion, NWEI—Dm4: Number of waveform events per insect—Phloem conditioning, NWEI—sgDm4: Number of waveform evens per insect—Single phloem conditioning not associated to phloem ingestion, WDEI—Dm4: Waveform duration event per insect—Phloem conditioning, t>1stDm4>1stDm5: Time from first phloem conditioning to first phloem ingestion, t>1stDm5: time to first phloem ingestion, WDEI—Dm5: Waveform duration event per insect—Phloem ingestion, WDI—Dm5: Waveform duration per insect—Phloem ingestion.

## Discussion

Probing behavior of *D*. *maidis* varied across hybrids tested, indicating that some of them exhibited traits related to plant resistance to insects, similarly to previous findings in other pathosystems. The reduction of duration of probes, together with an increase in their number for *D*. *maidis* mostly in DK390 is similar to previous findings in the leafhoppers *Nephottetix virescens* [21, 30] in rice, and *Graminella nigrifrons* [39] and *Psammotettix alienus* [40] in several hosts, the planthoppers *Nilaparvata lugens* [31, 33, 41] in rice, and *Delphacodes kuscheli* [42] in corn and oats, and the aphids *Myzus persicae* [43] in oilseed rape, *Rophalosiphum padi* [29] in barley, *Acirthosiphon pisum* [44] in pulse species, and *Aphis glycines* [45] in soybeans. This behavior is observed as insects reject non-hosts species or resistant genotypes before they contact phloem, implying that factors conferring plant resistance of the antixenosis type are located at the epidermis or mesophyll and force the insects to remove their stylets from the plants. Specific aspects of probing behavior related to resistance factors located at the epidermis, such as time to first probe and duration of first probe [29], were not found in this work with the hybrids tested because insects probed quickly (within seconds) after placed in contact with plants, and several brief probes (less than one minute) took place in all genotypes during the very first stages of insect-plant interaction. The shorter time spent on pathway waveform by insects probing on DK670 agree with the depiction of susceptible genotypes in other pathosystems, as in the leafhopper *Nephottetix* spp. [28] in rice, the planthoppers *N*. *lugens* [31] in rice and *D*. *kuscheli* [42] in corn, and the aphids *R*. *padi* [29], A. *glycines* [45, 46] and *A*. *pisum* [44] in several host species, indicating an antixenosis-based resistance factor located in mesophyll.

Xylem ingestion occurred in all the hybrids included in this work. As it has been already considered [25], this ingestion could be due to the need of insects to rehydrate after a dehydration period of about two hours during handling, before the start of the recording sessions. However, xylem ingestion at later stages of insect-plant interaction could be due to the need to rehydrate whenever the insects were not able to ingest from phloem, as it was mostly seen in the resistant hybrid DK390. This behavior was observed in *Nephottetix* spp. [28, 30] in rice, *G*. *nigrifrons* [39] in several hosts, *D*. *kuscheli* [42] in corn, and *R*. *padi* [29] in several hosts. Alternatively, other work in *N*. *virescens* [21] and *A*. *glycines* [46] found no differences in the time spent in xylem ingestion, an aspect that could be related to the insect species, the genotypes used, or the experiment setup as discussed above.

The time spent in phloem conditioning did not increase compared to the susceptible hybrid DK670, as in earlier studies of the leafhoppers *N*. *virescens* [30] and *G*. *nigrifrons* [39]. However, the planthopper *N*. *lugens* [31] and the aphid *M*. *persicae* [47], increased the total time spent in phloem conditioning in resistant genotypes. Probing behavior of *D*. *maidis* in DK72-10 and DK390 hybrids showed phloem-located resistance, although expressed differently. On one hand, the time from first phloem conditioning to first phloem ingestion was particularly longer in DK72-10, as described in the planthopper *D*. *kuscheli* [42] and the aphids *M*. *persicae* [43, 47], and *R*. *padi* [29]. Longer phloem conditioning events are considered necessary to prevent clogging of phloem cells and hence to maintain phloem ingestion [55]. On the other hand, the higher number and shorter duration of phloem conditioning events not associated with phloem ingestion in DK390 represent failed attempts to ingest from phloem. However, once this initial resistance was overcome, all the insects were able to ingest from phloem in both hybrids.

Insects having access to resistant hybrids spent less time ingesting from phloem as compared to DK670. This behavior has been found in other species, such as the leafhoppers *G*. *nigrifrons* [39] and *N*. *virescens* [21, 28, 30], the planthoppers *D*. *kuscheli* [42] and *N*. *lugens* [31, 41], and the aphids *A*. *glycines* [45, 46], *A*. *pisum* [44], *M*. *persicae* [43, 47] and *R*. *padi* [29]. These results indicate that factors conferring the antixenosis type of resistance prevented or reduced phloem ingestion. Another aspect to consider is the proportion of insects reaching phloem ingestion. While all the insects in our work ingested from phloem regardless the hybrid, only a portion of them reached phloem ingestion in other work of leafhoppers [30], planthoppers [31, 33, 42], or aphids [44, 46], due probably to insect and plant species used in each work.

Compared to the susceptible hybrid DK670, DK79-10 showed epidermis/mesophyll and phloem resistance. This hybrid showed no antixenosis in a previous work [22], so is either possible that probing behavior is a more precise tool for detecting traits conferring antixenosis than settling preference, or that this level of variation in probing behavior plays no role in antixenosis. The probing behavior in the hybrid DK72-10 was like DK79-10, having also phloem resistance by increasing the amount of time in phloem conditioning to attain phloem ingestion. This resulted in less time on this latter activity and increased xylem ingestion to avoid dehydration as seen in other species [28, 30, 46, 48]. Lastly, the hybrid DK390 altered *D*. *maidis* probing behavior at a higher level than DK79-10 and DK72-10, by disrupting probing at both epidermis-mesophyll and phloem conditioning levels. These results agree with previous work [22] finding antixenosis in both DK72-10 and DK390.

The primary current hypotheses of antibiosis and antixenosis against several hemipteran pests are: a) presence of toxins in either mesophyll or phloem tissues that are detected during insect probing and deter feeding [49–53], and b) blockage of phloem sieve tube elements due to the inability of proteins in insect saliva to avoid clogging and maintain phloem conditioning or ingestion [54–58]. Based on these hypotheses, if a toxin is the underlying resistance mechanism, this would be present only in DK72-10, which was the only hybrid that reduced insect survival [22], because DK390 did not. Alternatively, a resistance mechanism based on a failure to avoid phloem clogging could be a common mechanism for both hybrids. In addition, DK390 would also have a mesophyll-based resistance component of an unknown origin since no reduced survival was seen in this hybrid [22].

The proportion of insects reaching phloem is an aspect that allows us to infer the efficacy of plant resistance traits to insect vectors in excluding the transmission of persistently transmitted pathogens. In this sense, while all the insects in this study reached phloem phase in all genotypes tested, some insects probing on resistant genotypes in *N*. *virescens* [21, 28, 30], *P*. *alienus* [40] and *N*. *lugens* [31, 41] did not reach phloem. This is clearly related to the insect species and plant genotypes used in each study, but perhaps also to the length of the EPG recording session used in each work. In this study, a 10 h recording session was selected because previous research on this species [23, 27] indicated that insects needed this amount of time to ingest from phloem, even in susceptible genotypes. In this sense, studies using short recording lengths would overlook the ability of insects to reach phloem, even in resistant genotypes, to transmit pathogens accordingly. This aspect could be particularly useful for phloem-located pathogens in situations where the same genotype is deployed across large geographical areas, and the hosts “chosen” by insects would be mostly other plants of the same genotype.

While the characterization of the mechanisms of resistance to *D*. *maidis* in these hybrids is an open line for future research, another aspect to consider is how effectively they could prevent the inoculation of the pathogen *S*. *kunkelii* by *D*. *maidis*. In this sense, the time to first phloem ingestion (50% at six hours) coincided in time with hybrids DK390 and DK72-10 starting to be less preferred [22]. In this case, although these hybrids would prevent phloem ingestion, at least one event of phloem conditioning has likely taken place. Thus, the pathogen *S*. *kunkelii* could be successfully inoculated [27] before the insects leave the plant in search of more suitable hosts. Furthermore, insects moving to other plants of the same genotype would repeat this probing behavior, inoculating the pathogen to other plants. It could be possible that the inoculated pathogen is blocked by phloem proteins that promote phloem clogging, and so it would not be able to spread and induce a systemic infection in the plant. The authors are not aware of any research in this direction at the present time, and hence this also may be an area of further research. Mesophyll-located resistance factors could also be a promising tool to prevent inoculation of *S*. *kunkelii*, since they operate at early stages of insect-plant interaction, before insects make their first contact with phloem. This could potentially reduce the inoculation efficiency of *S*. *kunkelii* and perhaps explain the slightly lower inoculation efficiency in DK390 (84%) compared to the other hybrids (100%) using five inoculative insects per plant [22]. This is also another potential area of research, which could benefit from the use of EPG recording with inoculative insects, and also help to dissect either *D*. *maidis* or *S*. *kunkelii* as the target of resistance in maize genotypes, as it has been discussed before [19, 20, 22].

This work provided a detailed characterization of maize resistance mechanisms to the corn leafhopper *D*. *maidis*. Sources of resistance were detected in mesophyll and phloem tissues, providing a tool to identify and combine sources of resistance, reducing the negative impact of corn stunt in field. Further research will aim to identify genes related to this resistance, and to further characterize their impact in reducing the transmission efficiency of *S*. *kunkelii*.

## Supporting information

S1 Fig Progression of phloem conditioning (Dm4) into phloem ingestion (Dm5) of *D*. *maidis* insects with access to maize hybrids (retrieved from raw data in Carpane et al. 2011 [27]. Inlets: A, C-F: phloem conditioning, B: “spikes” of high amplitude and irregular shape, G: end of phloem conditioning and transition to early phloem ingestion, H: phloem ingestion.
(TIF)

S1 Raw data (XLSX)
