# Supplementary figures and images for: Probing behavior of the corn leafhopper Dalbulus maidis on susceptible and resistant maize hybrids
Source: PLoS One. 2022 May 31;17(5):e0259481. doi: 10.1371/journal.pone.0259481 (PMC9154111; doi:10.1371/journal.pone.0259481)

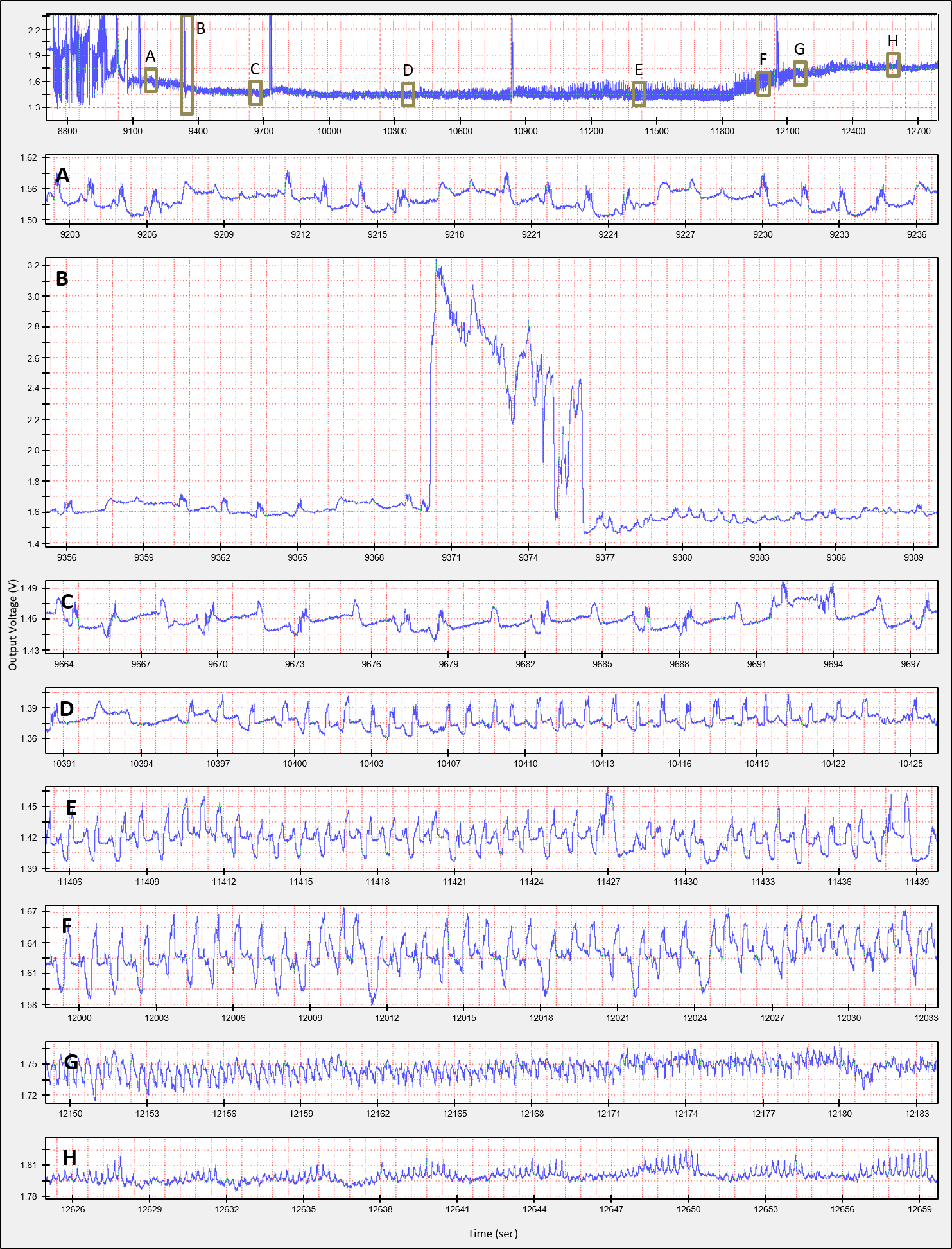

Supplement: S1 Fig — Inlets: A, C-F: phloem conditioning, B: “spikes” of high amplitude and irregular shape, G: end of phloem conditioning and transition to early phloem ingestion, H: phloem ingestion. (TIF) [file pone.0259481.s001.tif]
